# Measurement of femoral head penetration in polyethylene using a 3-dimensional CT-scan technique

**DOI:** 10.3109/17453674.2010.519163

**Published:** 2010-10-08

**Authors:** Eric Vandenbussche, Mohammed Saffarini, Ulrich Hansen, Fabienne Taillieu, Céline Mutschler, Bernard Augereau, Thomas M Gregory

**Affiliations:** ^1^Department of Orthopaedic Surgery, Université Paris Descartes, Assistance Publique – Hôpitaux de Paris, Hôpital Européen Georges Pompidou, Paris, France; ^2^Department of Radiology, Université Paris Descartes, Assistance Publique – Hôpitaux de Paris, Hôpital Européen Georges Pompidou, Paris, France; ^3^Department of Mechanical Engineering, Imperial College, London, UK; ^4^Department of Health Science Research, Qatar Science and Technology Park, Qatar Foundation, Doha, Qatar

## Abstract

**Background:**

Current techniques for measuring in vivo polyethylene wear suffer from a range of problems, resulting in an unacceptable lack of repeatability and/or insufficient accuracy when they are used to measure the low wear rates associated with new, highly crosslinked polyethylene. We describe an improved CT method for measurement of 3D femoral head penetration in PE acetabular cups that has sufficient accuracy and repeatability to allow assessment of the wear potential of modern implants.

**Method:**

The accuracy and repeatability of the CT-scan method was determined by blindly repeating measurements on a precisely calibrated 28-mm prosthetic head and by comparing them with direct metrological measurements on 10 acetabular specimens with in vitro wear from machining, and on 8 explanted acetabular specimens with in vivo wear.

**Results:**

The intra- and interobserver errors in femoral head diameter were 0.036 mm (SD 0.044) and 0.050 mm (SD 0.022), respectively. CT estimated femoral head penetration in both all-poly and metal-backed acetabular components with accuracy ranging from 0.009 to 0.245 mm (mean 0.080; SD 0.067).

**Interpretation:**

We found that the CT method is rapid, is accurate, and has repeatability and ease of availability. Using a slice thickness of 0.0625 mm, this method can detect wear—and also the threshold for the wear rate that causes osteolysis—much earlier than previous methods.

The consequences of polyethylene (PE) wear remain a principal cause of long-term failure in total hip arthroplasty (THA). The continued development of new, highly crosslinked PE is allowing improvement in in vivo methods of wear measurement. The classic manual methods have gradually been replaced by more modern techniques with higher accuracy and repeatability, essentially based on digitization of X-ray images and computational data processing (Martell and Berdia 1997, Shaver et al. 1997, Pedersen et al. 1998, Phillips et al. 2002, Martell et al. 2003, The et al. 2006) Complementary sagittal X-rays improve results and enable calculation of 3-dimensional (3D) wear, but the variable quality of X-ray images limits the repeatability of these methods (Martell et al. 2003, Bragdon et al. 2005).

Most of these methods determine 2-dimensional (2D) wear and their accuracy ranges from 0.010 to 0.500 mm (95% confidence band: 0.200–0.400 mm) (The et al. 2006). Despite the claimed accuracy, study results do not always match direct measurements on samples explanted at revision surgery (Barrack et al. 2001). The existing techniques are all associated with complex problems of relative pelvic position, X-ray beam centering, and errors in radiographic magnification.

Computed tomography has been used by numerous authors to study periprosthetic osteolysis and to correlate it with PE wear estimated by radiographic measurements (Looney et al. 2002, Kitamura et al. 2005). The more recent papers on this topic refer to femoral head penetration in the acetabular cup, which includes both bedding-in due to plastic deformation and the true wear (Sychterz et al. 1997, Dowd et al. 2000, McCalden et al. 2005, Bragdon et al. 2006, 2007). Use of the CT technique for calculation of PE wear has also been described (Olivecrona et al. 2003, 2005, Jedenmalm et al. 2008). These studies established the principal use of the CT method for measurement of wear but showed that there were various shortcomings, thus limiting its use. Notably, the CT technique was only shown to be useful when evaluating metal-baked implants and was only validated against a small number of such implants. Perhaps more importantly, the accuracy reported was only 0.6 mm, i.e. the same accuracy as that of radiographic techniques, and it was possibly inadequate for detection of the expected low wear rates of less than 0.1 mm/year seen with the new highly cross-linked polyethylenes (McCalden et al. 2005).

Here we describe an improved CT method for measurement of 3D femoral head penetration in PE acetabular cups. We have determined the accuracy and repeatability of this technique.

## Methods

The first step of the study was to determine the intrinsic accuracy and repeatability of CT measurements on a precisely calibrated prosthetic femoral head. The specimen was digitized 10 times blindly by the same observer and then by 10 other observers to calculate intra- and interobserver variance. The second step was to determine the accuracy of CT measurements of femoral head penetration in different acetabular cups with unidirectional PE wear (implants with in vitro wear from precise machining). The last step was to determine the accuracy of CT measurements of femoral head penetration in different acetabular cups with possible multidirectional PE wear (implants worn in vivo and explanted at revision surgery).

The specimens used were considered as 3 groups (Table 1). Group A comprised a single cobalt-chromium (CoCr) femoral head (Zimmer Inc., Warsaw, IN). The implant was unused and had a nominal diameter of 28 mm and a real diameter 27.957 mm as measured using a Crysta Apex C574 coordinate measurement machine (Mitutoyo Corp, Waltham, MA). Group B consisted of 10 acetabular components (and matching femoral heads) that were artificially worn by machining at 45° to their equatorial planes using an Integrex 200-III 6-axe milling machine (Mazak Corp, Florence, KY). 5 of the acetabular components were cemented all-poly Acoplot implants (Zimmer) and the 5 others were uncemented metal-backed PE Pressfit Kappa Acora implants (Zimmer). Group C consisted of 8 acetabular components (and matching femoral heads) of different models that were worn extensively in vivo and explanted during revision surgery. 4 of the acetabular components were cemented all-poly implants and the 4 others were uncemented metal-backed PE implants.

**Table 1.** Materials used in this study

Group	Components	Quantity	Material	Model	Manufacturer	Articular diameter (mm)	Notes
A	Femoral Head	1	CoCr		Zimmer	28 (exact) 27.957	Unused implant with exact diameter determined by coordinate measurement machine
B	Acetabular Cups	5	All-PE	Acoplot	Zimmer	22.2	All implants were worn artificially by machining the articular surface
		5	MB-PE	Kappa Acora	Zimmer	22.2	
C	Acetabular Cups	2	All-PE	PSA	Stratek	26	All implants were worn naturally in vivo and explanted at revision surgery
		2	All-PE	Charnley	SEM	32	
		2	MB-PE	PSA	Stratek	26	
		1	MB-PE	Screw Ring	Benoist Girard	28	
		1	MB-PE	Spotorno Pressfit	Implex Corp	28	

CoCr = cobalt-chromium; MB = metal-backed; PE = polyethylene

**Figure 1.**
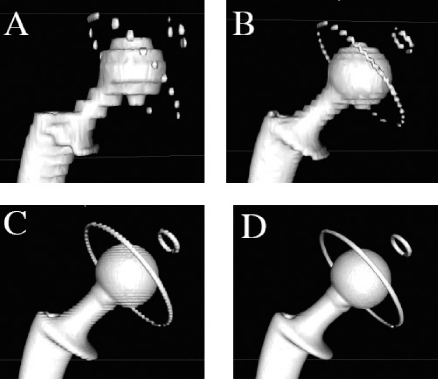
3-dimensional image reconstructions of a CT-scanned all-poly acetabular component assembled with its matching femoral stem and 28-mm diameter head. The images were produced using the surface rendering function in OsiriX and illustrate the ‘partial voluming effect’ that increases with thickness of CT section slice: A. 5 mm; B. 2.5 mm; C. 1.25 mm; D. 0.625 mm.

The acetabular components of groups B and C were each measured directly using a Crysta Apex C574 coordinate measurement machine to accurately record the extent of femoral head penetration. Direct measurement of femoral head penetration was considered to be the gold standard, and we intended to use the direct measurements to evaluate the accuracy of subsequent CT measurements. Each acetabular component was assembled with its matching metallic femoral head in the position of maximum penetration and affixed using adhesive tape. All specimens (groups A, B, and C) were scanned following an identical protocol using a 64-slice Multi-Detector Scanner (General Electric, Waukesha, WI). The CT scans (image size: 512 × 512 pixels; field of view: 36 cm) were taken with a contiguous thickness of 0.625 mm, with settings of 12 kV and 70–120 mA. Each CT scan contained 200–350 DICOM images and was recorded on a separate CD-ROM. We used image processing software dedicated to DICOM images, OsiriX (open-source software; www.osirixviewer.com), to generate 3D reconstructions of each CT scan (Rosset et al. 2005). The software benefits from the extremely fast and optimized 3D graphic capabilities of the OpenGL graphic standard, optimized to exploit any available hardware graphic accelerator boards. The 3D surface reconstructions were created using the surface rendering function in OsiriX with software settings optimized for metal (pixel value, 2,000; high resolution, 0.50; smooth iterations, 80). We noticed that the quality of 3D surface reconstructions deteriorated progressively with the thickness of the CT slice used (1.25, 2.5, or 5 mm), producing stepped surfaces (Figure 1). Thus, to avoid measurement errors from such artifacts, all image reconstructions were made using the native section scans of 0.625 mm thickness.

**Figure 2.**
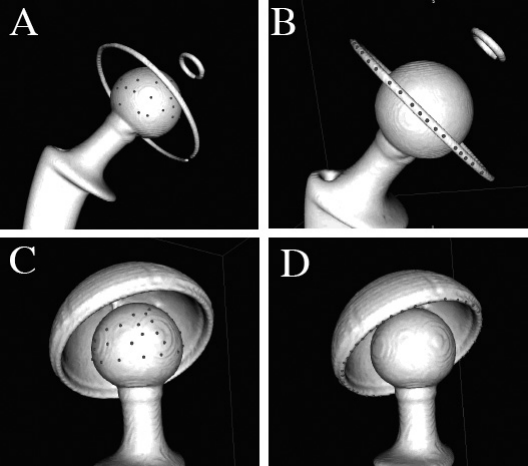
Digitization of points on 3-dimensional image reconstructions of CT- scanned implants. A and B. An all-poly acetabular component assembled with its matching femoral stem and 28-mm diameter head. C and D. A metal-backed PE acetabular component assembled with its matching femoral stem and 32-mm diameter head.

For each of the 3D image reconstructions, we digitized the femoral head with 40 points evenly dispersed on the spherical portion, and digitized the acetabular component with 40 points evenly dispersed on the peripheral metal ring (all-poly cups) or on the flat rim of the metal shell (metal-backed cups). The femoral head is therefore represented by a cloud of points that lie on a unique sphere, and the acetabular component by a succession of points that lie on a unique circle in space (Figure 2). While digitizing a point on a metallic component, OsiriX placed the point precisely on the metal surface in the 3D view with a spatial accuracy of 0.001 mm. Note that the 28-mm diameter femoral head (group A) was used to study the intra- and interobserver accuracy and repeatability of our method. Thus, the femoral head digitization was blindly repeated 10 times by the same observer (EV) and then by 10 other surgeons at our center.

**Figure 3.**
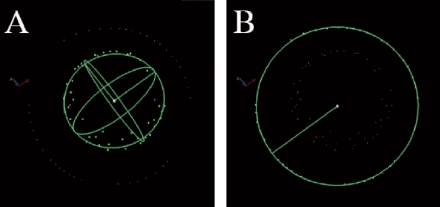
Calculation using 3DReshaper, by the method of least squares, of the center and diameter of a spherical femoral head (panel A) and the center and diameter of a circular rim of a metal-backed acetabular component (panel B).

The coordinates of digitized points were exported from OsiriX to a file as comma-separated variables (CSVs). Geometric analysis software, 3D Reshaper (Technodigit, Gleizé, France), was used to deduce the coordinates of the articular head center by fitting a sphere to its point cloud, and the center of the acetabular component by fitting a circle to its point array, in both cases using the method of least squares (Figure 3). The point coordinates were manipulated further in spreadsheets using Microsoft Excel to calibrate all coordinates to the known femoral head diameter and to deduce the head-cup eccentricity with reference to the engineering drawings of each acetabular component.

## Results

### Group A

For the CoCr femoral head of real diameter 27.957 mm, the mean intraobserver diameter was 27.993 (27.893–28.061) mm (SD 0.044) and the mean interobserver diameter was 28.007 (27.984–28.061) mm (SD 0.022) (Table 2). The real errors of mean intra- and interobserver diameters were therefore 0.036 mm and 0.050 mm, respectively, with a standard deviation of less than 0.050 mm. The mean intra- and interobserver coordinates of the head center also had a standard deviation of less than 0.050 mm on the x- and y-axes, but up to 0.096 mm for the z-axis.

**Table 2.** Accuracy and repeatability of intra- and interobserver CT measurements on unused CoCr femoral heads

Group	Measurment			Mean	SD	Minimum	Maximum	SEM	95% CI	Real error
A	Diameter	Mean intraobserver (mm)		27.993	0.044	27.893	28.061	0.031	0.001	0.036
	Diameter	Mean interobserver (mm)		28.007	0.022	27.984	28.061	0.016	0.001	0.050
	Center	Mean intraobserver	x (mm)	0.000	0.038	–0.075	0.058	0.030	0.0007	
			y (mm)	0.000	0.021	–0.027	0.032	0.018	0.0004	
			z (mm)	0.000	0.096	–0.183	0.164	0.065	0.0019	
	Center	Mean interobserver	x (mm)	–0.032	0.044	–0.110	0.079	0.031	0.0008	
			y (mm)	0.025	0.025	–0.012	0.048	0.018	0.0005	
			z (mm)	–0.071	0.065	–0.183	0.090	0.053	0.0013	

SD: standard deviation; SEM: standard error of the mean; CI: Confidence interval; Real error: the difference between the head diameter measured and the real head diameter (27.957mm) Mean intraobserver measurements from 10 repeatedly blinded CT measurements by the same senior surgeon (EV) Mean interobserver measurements from 10 repeatedly blinded CT measurements by 10 different surgeons.

### Group B

For the 10 implants that were worn artificially by machining, the real mean error in femoral head penetration measurements using CT compared to direct measurements was 0.101 (0.011–0.245) mm (Table 3). The real mean error for all-poly implants was 0.118 mm, which was close to the real mean error for metal-backed PE implants (0.083 mm).

**Table 3.** Measurments of 3D femoral head penetration in explanted and machined acetabular cups

Group	Component number	Material	Model	Manufacturer	Articular diameter (mm)	3D femoral head penetration (mm)		
						Direct	CT	Real error
B	1	All-PE	Acoplot	Zimmer	22.2	0.993	1.183	0.190
	2	All-PE	Acoplot	Zimmer	22.2	2.001	1.990	-0.011
	3	All-PE	Acoplot	Zimmer	22.2	2.994	3.076	0.082
	4	All-PE	Acoplot	Zimmer	22.2	4.001	3.939	-0.062
	5	All-PE	Acoplot	Zimmer	22.2	4.995	4.750	-0.245
	6	MB-PE	Kappa Acora	Zimmer	22.2	1.004	0.924	–0.080
	7	MB-PE	Kappa Acora	Zimmer	22.2	1.994	2.006	0.012
	8	MB-PE	Kappa Acora	Zimmer	22.2	2.984	2.802	–0.182
	9	MB-PE	Kappa Acora	Zimmer	22.2	4.002	4.119	0.117
	10	MB-PE	Kappa Acora	Zimmer	22.2	10.493	10.469	–0.024
C	11	All-PE	PSA	Stratek	26	5.475	5.448	–0.027
	12	All-PE	PSA	Stratek	26	2.128	2.020	–0.108
	13	All-PE	Charnley	SEM	32	3.126	3.203	0.077
	14	All-PE	Charnley	SEM	32	3.229	3.181	–0.048
	15	MB-PE	PSA	Stratek	26	3.130	3.042	–0.088
	16	MB-PE	PSA	Stratek	26	4.071	4.080	0.009
	17	MB-PE	Screw Ring	Benoist Girard	28	3.943	3.920	–0.023
	18	MB-PE	Spotorno Pressfit	Implex Corp	28	2.240	2.178	–0.062

MB: metal-backed; PE: polyethylene

### Group C

For the 8 acetabular explants with natural in vivo wear, the real mean error in femoral head penetration measurements using CT compared to direct measurements was 0.055 (0.009–0.108) mm (Table 3). The real error for all-poly implants (mean 0.065 mm) was close to the real error for metal-backed PE implants (mean 0.046 mm).

The absolute real error of femoral head penetration for groups B and C was 0.080 mm. We observed that the absolute real error of femoral head penetration was lower for group C (mean 0.055 mm) than for group B (mean 0.101 mm). We also noticed that the absolute real error of femoral head penetration was in general slightly higher for all-poly implants (mean 0.094 mm) than for metal-backed PE implants (mean 0.066 mm). It was not possible to determine whether differences in accuracy between implant types were statistically significant, because of the small sample size and because the errors were close to the inherent accuracy of the measurement technique.

## Discussion

The 3D reconstruction of CT scans described here allows the penetration in PE acetabular implants to be measured with an accuracy of 0.080 mm and a repeatability of ± 0.040 mm.

This is comparable to recently reported accuracies of radiographic techniques, which ranged between 0.010 and 0.500 mm (95% confidence band: 0.200–0.400mm) (The et al. 2006). However, it is noteworthy that radiographic techniques determine 2D penetration from frontal radiographs whereas our method determines a 3D penetration vector (magnitude and direction), which is more realistic. Also, all radiographic techniques are subject to complex issues of pelvic positioning, beam centering, and errors in radiographic magnification. These issues jeopardize duplication of the technique (Shaver et al. 1997, Kitamura et al. 2005) and study results do not always agree with direct measurements of the explanted acetabular cup at the time of revision surgery (Barrack et al. 2001).

The accuracy and repeatability of our results are achieved partly by digitization of a large number of points on each component. Although mathematically, 4 points are sufficient to define the center and diameter of a sphere and 3 points are sufficient to define the center and diameter of a circle, we chose to digitize 40 points both on the femoral head and on the acetabular cup. The large number of data points helped to cancel out errors due to irregularities of reconstructed surfaces and to minimize errors induced by the CT scanner, which has a spatial accuracy of 0.001 mm in coordinates on the x- and y-axes but assigns the z-coordinate to true CT section, spaced 0.625 mm apart. The latter explains why the standard deviation is greater in the z-coordinate when calculating the head center (Table 2).

The CT method for measurement of femoral head penetration is based solely on 3D analysis of CT scans. The method requires availability of raw scan data without reconstruction, with section slices of 0.625 mm thick, presetting of parameters in the OsiriX imaging software, and availability of implant engineering drawings with clear dimensions. The method may seem complex, but it relies on simple principles, is easily accessible, and does not require expensive equipment. The technique is applicable to both all-poly and metal-backed PE acetabular implants, unlike the computerized radiographic techniques—which are only applicable with sufficient accuracy to metal-backed components (Barrack et al. 2001). In addition to femoral head penetration, the CT method can determine the spatial orientation of the acetabular implant in vivo with respect to the frontal pelvic plane, if only three anatomic landmarks are digitized on the pelvis (Lewinnek et al. 1978). Simple software can then calculate the cup anteversion and inclination angles as commonly done in navigation (Vandenbussche et al. 2007, 2008).

Olivecrona et al. (2003) and Jedenmalm et al. (2008) described a technique for diagnosis of acetabular cup wear using computed tomography. They reported that the CT technique had a relatively low accuracy of 0.6 mm (Olivecrona et al. 2005). This shortcoming and others were acknowledged by these authors, but have been addressed and solved in this paper. Their method was restricted to metal-backed implants. In our study, both all-poly and metal-backed implants were analyzed and the accuracy reported is of the same magnitude. Thus, we have also established the use of the method for all-PE implants.

These previously published papers analyzed wear based on only 8 explants, which may not have been adequate to establish confidence in the use of the CT method. Our study included 18 implants and it has given more confidence in the use of the CT method for the analysis of wear.

In addition, our method resulted in an accuracy of 0.08 mm whereas Olivecrona et al. (2005) reported an accuracy of 0.6 mm, which is of the same magnitude as the radiographic methods. This improvement in accuracy is crucial for the early detection of wear, as mentioned below. We suspect that this improvement is directly associated with the slice thickness chosen: 0.625 mm in our study as compared with 1.25 mm in the earlier papers. This is consistent with the gradual deterioration of the 3D surface reconstruction observed when using thick slices (1.25, 2.5, and 5 mm), as illustrated in Figure 1.

In summary, the accuracy of the CT technique described in this paper is 0.08 mm and the repeatability is 0.04 mm. This accuracy is much better than for other methods where accuracies of between 0.2 and 0.4 mm for radiographic techniques and 0.6 mm for the CT technique have been reported. In a recent overview (McCalden et al. 2005), the clinical wear rate of polyethylene was reported to be in the range of 0.1 to 0.4 mm annually. With an accuracy of 0.08 mm, it will be possible to detect wear much earlier than with previous methods. These earlier methods would have to wait for many years before being able to determine whether an implant has wear. This is particularly relevant for the new, highly crosslinked PE with wear rates of below 0.1 mm/year. Also, the threshold for the wear rate that causes osteolysis is reported to be 0.1 mm (Dumbleton et al. 2002, McCalden et al. 2005). Thus, the CT method we describe can quickly detect whether this important threshold has been violated whereas such a decision cannot be made for many years with other methods.

Our method has a number of limitations if applied to in vivo measurements on THA. Firstly, although not refuted in the literature, it may seem erroneous to measure wear or penetration with the patient not weight-bearing (Smith et al. 1999, Digas et al. 2003). Numerous authors have, however, reported that there is no difference between the wear measured on supine radiographs and that measured on standing radiographs (Martell et al. 2000, Moore et al. 2000, Digas et al. 2004, Bragdon et al. 2006). Secondly, both types of acetabular implant may introduce a source of error to the measurements: (1) with all-poly acetabular implants, measurements rely on the integrity of the metallic wire ring, which may be deformed during cup impaction, as evident on radiographs with ellipsoid rings or non-union at ends. Whereas the wire rings in Charnley-type cups are clearly visible (stainless steel, diameter 1.0–1.2 mm), they tend to be displaced or deformed more easily because they are placed in a relatively wide groove. The wire rings in Muller-type cups are less visible (titanium, diameter 0.8 mm), but tend to be more robust because they are press-fitted in an undersized groove to inhibit movements; (2) with metal-backed PE implants, the beam-hardening artifact can alter the rendered sphericity of the metallic femoral head therein. This error is exacerbated in cases where the metal shell is thick, has a small outer diameter, or is made from high radiodensity metal such as cobalt-chromium. We have not observed such cases in our study, however, because all metal-backed shells were made of titanium, which has a lower relative radiodensity.

CT is an expensive diagnostic tool that exposes patients to high doses of radiation. The radiation dose of a CT scan limited to the pelvis (5 mSv, 500 mrem) is only one-fifth of that reported by Brenner et al. to have a proven link to cancer (Brenner and Hall 2007). Also, this risk is limited in elderly patients, who represent most of the arthroplasty recipients. However, the benefit to the patient of the information gained in measuring the acetabular wear probably does not outweigh the risk of radiation exposure. Thus, we cannot advocate the use of CT scan for the sole purpose of wear measurement, even though it overcomes many of the limitations of standard radiographs.

CT is the modality of choice, however, for assessment of periprosthetic osteolysis. Then, if THA CT scan is requested, the 3D reconstruction protocol described in this paper for acetabular wear can be used to gain additional information without additional radiation exposure.

Our study has shown that the CT method is rapid, accurate, repeatable, and easily available. This method, using a slice thickness of 0.0625 mm, can detect wear—and the threshold for the wear rate that causes osteolysis—much earlier than previous methods. This latter capability is particularly essential when trying to evaluate the clinical performance of new, highly crosslinked polyethylene.
